# Impact of a Nutrition Education Intervention on Salt/Sodium Related Knowledge, Attitude, and Practice of University Students

**DOI:** 10.3389/fnut.2022.830262

**Published:** 2022-02-25

**Authors:** Leila Cheikh Ismail, Mona Hashim, Amjad H. Jarrar, Maysm N. Mohamad, Rameez Al Daour, Radhiya Al Rajaby, Sara AlWatani, Amna AlAhmed, Shaikha Qarata, Fatima Maidan, Sheima T. Saleh, Lily Stojanovska, Ayesha S. Al Dhaheri

**Affiliations:** ^1^Department of Clinical Nutrition and Dietetics, College of Health Sciences, University of Sharjah, Sharjah, United Arab Emirates; ^2^Nuffield Department of Women's and Reproductive Health, University of Oxford, Oxford, United Kingdom; ^3^Department of Nutrition and Health, College of Medicine and Health Sciences, United Arab Emirates University, Al Ain, United Arab Emirates; ^4^Institute for Health and Sport, Victoria University, Melbourne, VIC, Australia

**Keywords:** educational intervention, dietary salt, dietary sodium, knowledge retention, university students

## Abstract

**Background:**

Salt reduction strategies help reduce the risk of cardiovascular diseases (CVDs) by reducing high blood pressure. This study aimed to assess salt related knowledge, attitude, and practices (KAP) before and after administering an evidence-based nutrition education workshop.

**Methods:**

Ninety non-medical university students were recruited to investigate KAP related to dietary salt intake. The KAP components were assessed before, immediately after, and 4-weeks after administering an evidence-based educational workshop and leaflet.

**Results:**

Knowledge and attitudes related to salt improved significantly immediately post-intervention but were not fully retained after 4-weeks. Five of the 13 evaluated practices improved after 4-weeks: trying to buy low-salt foods increased from 10 to 19% (*P* = 0.022), rarely adding salt to food during cooking increased from 5 to 16% (*P* = 0.019), rarely adding salt to food at the table increased from 29 to 42% (*P* = 0.011), tried to reduce salt intake increased from 26 to 41% (*P* = 0.014), and tried to use spices to reduce salt increased from 31 to 45% (*P* = 0.044).

**Conclusions:**

The educational intervention had a positive impact on salt-related knowledge, attitudes, and practices, but the effect was not fully retained on the long-term. Periodic educational interventions should be considered to refresh knowledge and reinforce practices.

## Introduction

Non-communicable diseases (NCDs) cause premature death in over 15 million people between the ages of 30–70 years annually (1). Cardiovascular diseases (CVDs) contribute to most of the NCDs deaths followed by cancers, respiratory diseases, and diabetes (2). Measurable global policies for the averting of CVDs are required to reduce exposure of populations to risk factors as well as early detection and treatment at individual levels. Interventions targeting the reduction of the four main risk factors of cardiovascular diseases: tobacco use, harmful alcohol consumption, unhealthy diet, and physical inactivity, could prevent much of the morbidity and mortality caused by NCDs (3). Moreover, high dietary intake of salt/sodium is associated with elevated blood pressure and is one of the major contributors of premature deaths from cardiovascular diseases globally (4). The recent modeling from the Global Burden of Disease data estimates that globally the average salt intake in adults is 14 g per day (5).

Considering this, in 2013 the World Health Assembly (WHA) committed to nine global voluntary targets to reduce NCDs and a 30% relative reduction in a mean population intake of salt/sodium by 2025 (6). To achieve this, the action plan by the World Health Organization (WHO) recommends adults to consume <5 g of salt or 2 g of sodium per day (7). Supporting the action plan of the WHO, the World Action of Salt, Sugar and Health (WASSH), was established in 2005 to encourage sodium reduction worldwide and it currently has expert members in 100 countries (8). Countries around the world were encouraged to implement the action plan of the WHO through five key components: surveillance, product reformulation, standardized food labeling, knowledge, and environment (9). Globally there has been an increase in the number of countries implementing different approaches. But none have yet met the targeted 30% relative reduction in salt intake from baseline (10). Reformulation strategies are likely to be more effective in countries where a large proportion of dietary salt comes from packaged foods and food prepared outside the home, whereas salt substitution may be more effective in countries where there is extensive use of discretionary salt.

Current salt intakes in the Eastern Mediterranean Region (EMR) are very high with an average intake of more than 12 g per person per day, which is more than double the recommended level by the WHO (11). Countries of the Gulf Cooperation Council (GCC) started taking actions to develop framework of salt reduction action plans and some were able to achieve 20% salt reduction in bread (12). Bread is a popular staple food in the GCC countries, and it is one of the main contributors of salt in the diet (12). Salt reduction interventions in the Eastern Mediterranean region focus on awareness campaigns to assist consumers in making informed choices and product reformulation to reduce salt content in processed foods (11).

In the United Arab Emirates (UAE), NCDs account for more than 75% of deaths each year, with CVDs contributing to about half of the total deaths (13). Evidence shows that hypertension is more prevalent among adults; with a prevalence rate of 53% and 47% among the females and males, respectively, in the UAE (13). Notwithstanding, hypertension and heart disease prevalence is increasing among younger population and therefore salt reduction strategies should be addressed with serious measures (14). A recent study among adult residents of the UAE measured 24-h urinary sodium excretion and revealed that about two thirds of the participants were significantly exceeding sodium recommendation dose of a mean of ~2,700 mg/day (15). Moreover, a study among the university students in the UAE indicated an alarming rate of 89% of the students exceeding the recommendation for dietary sodium intake with a mean dose of 3,571 mg/day (16). The Ministry of Health and prevention (MOHAP) in the UAE is leading the salt intake reduction strategies and activities focusing on product reformulation and empowering consumers with the knowledge needed to make healthier choices (17).

The available literature indicates low salt/sodium related knowledge and poor attitudes and practices toward salt (18–21). Similar findings were also reported in cross sectional studies in the UAE (15, 16). An intervention study was conducted on cardiac patients in Lebanon to assess their knowledge and attitudes toward salt/sodium consumption before and after distributing an educational leaflet, and it concluded a favorable impact on salt-related knowledge (22). Similarly, a salt reduction program implementation in 120 villages around China suggested positive effect on salt-related knowledge and attitudes of the study sample, which in turn contributed to a reduction in salt consumption (23).

That being the case, increasing the population knowledge and investing in intervention strategies related to salt reduction may assist in reducing the risk of high blood pressure and act as a preventative measure against CVDs.

Previously gathered baseline data on knowledge, attitudes and practices related to salt/sodium consumption among the university students in the UAE identified several salt-related knowledge gaps and engaging in unfavorable attitudes and practices (16). Moreover, the 24-h dietary recall revealed a high percentage of students exceeding the recommended level of dietary sodium intake (16). Based on this population specific evidence, the current educational intervention study was designed to determine the impact of a nutrition education intervention on salt-related knowledge, attitudes, and practices among the UAE university students.

## Materials and Methods

This study was approved from the University of Sharjah Research Ethics Committee (UOS-REC) reference number: REC-19-04-15-01-S. This study was conducted according to the stated principles in the Declaration of Helsinki (24). A written informed consent form was obtained from all participants.

### Participant Recruitment

**Figure 1:**
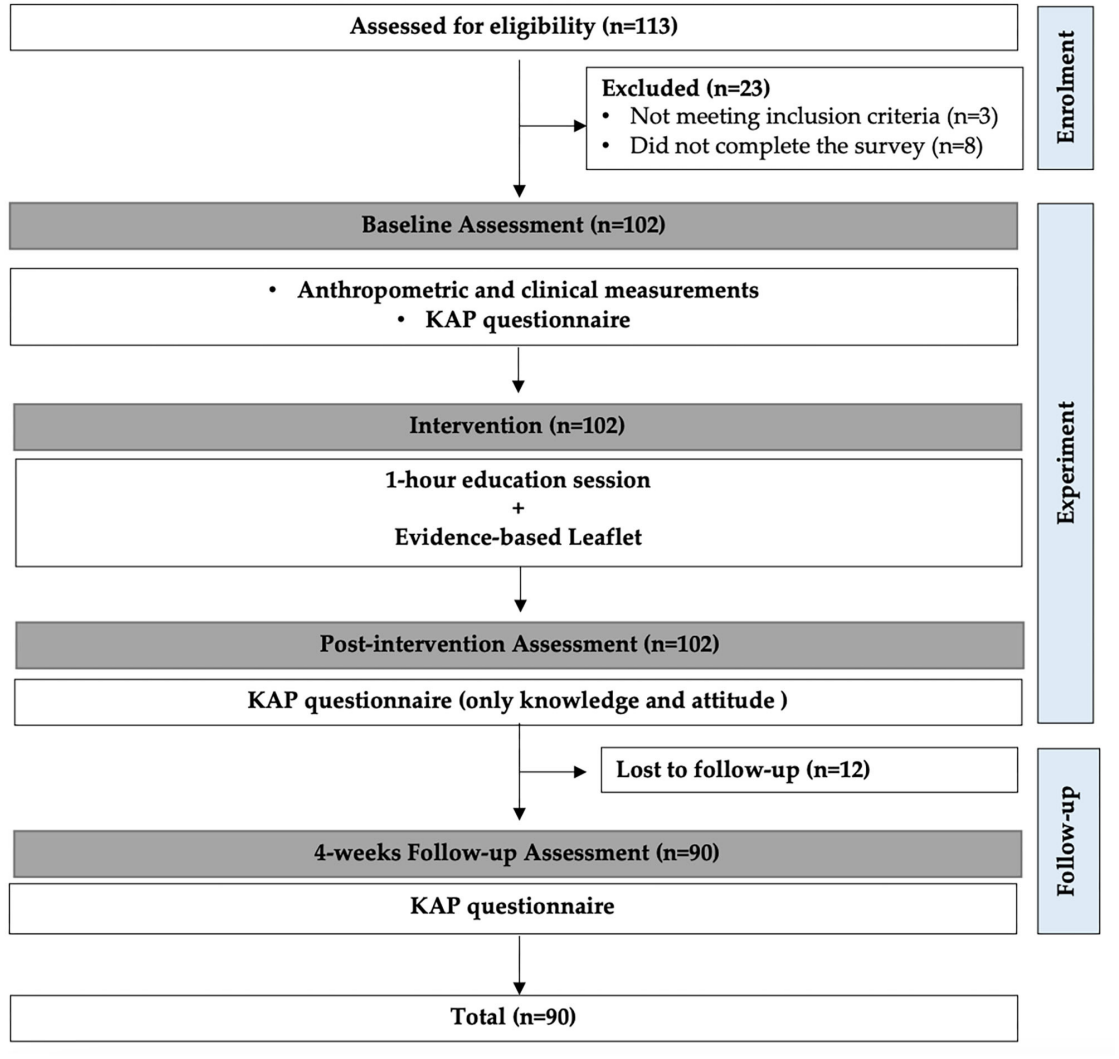
Flow of participants and study design.

An experimental (one group pre-test post-test) research design was used on a sample of students from the University of Sharjah (UOS), UAE, during the academic year of 2018/2019. Non-medical students from Applied Sciences and Humanities Colleges (without prior knowledge of cardiovascular diseases) were invited to participate in the study through email invitations (*n* = 1,200). About 113 students, aged 18–24 years old showed interest in the study, out of which, only 90 students met the following inclusion criteria: not having a history of hypertension, coronary artery disease or heart failure and no prior education about cardiovascular diseases. Three were previously diagnosed with hypertension or cardiovascular disease (excluded due to prior knowledge of cardiovascular diseases), eight did not complete the study survey, and twelve were lost to follow-up (Figure 1).

The sample size calculation was performed using G^*^Power software, version 3.01 (Franz Faul, Christian-Albrechts-Universität Kiel, Kiel, Germany) (25) for the two dependent groups (McNemar test). The calculation revealed the need for a sample of 57 participants to be included in the study. The level of salt related knowledge among students was assumed to be similar to that found in a previous study conducted among UOS students (16) and the significance level was set at *p* < 0.05 and the power was 0.80. Considering drop-out factors of 20%, the sample size was inflated to be 69.

### Pre-intervention Assessment

Anthropometric measurements including height and weight were determined. Height was measured without shoes and recorded to the nearest 0.5 cm. Weight was measured with light clothing on and recorded to the nearest 0.1 kg. Measurements were performed using a calibrated medical scale and stadiometer (SECA 284; Seca, Hamburg, Germany). Body mass index (BMI) was calculated by dividing weight (kg) by the height (m) squared (kg/m^2^) and then classified based on WHO cutoffs (26).

A pre-intervention bilingual (English and Arabic) multicomponent, self-administered questionnaire was administered to students. It included questions on socio-demographic, knowledge, attitudes, and practices (KAP) toward salt/sodium consumption. This questionnaire was previously validated in student's population and adult population aged 20–60 years old in the UAE (15, 16). The details of the KAP questionnaire have been described elsewhere (15, 16). In brief, it included a socio-demographic section (age, gender, nationality, college, residence, and choice of meals), 29 questions on salt/sodium related knowledge, 5 questions on attitudes, and 13 questions on practices. The full version of the questionnaire is available a Supplementary File.

### The Intervention

After completing the pre-intervention assessment, students attended an educational workshop on the importance of salt reduction. The educational material was given to participants through a single 1-h interactive session via power point presentation followed by 30 min activities to ensure involvement of the participant.

The 1-h educational presentation included information on definition of salt, differences between salt and sodium, salt intake recommendation compared to average salt consumption, food labels reading to identify low, medium, and high salt foods, strategies for salt intake reduction along with meal alternatives ideas, and information about salt related diseases.

The 30 min activities post presentation included guessing games such as estimation of sodium amount in various provided food samples, categorization of some commonly consumed dishes to low and high sodium content, and differentiation between two salad dishes: one contained salt in the dressing and the other one with spices and herbs substituting the salt. Toward the end of the session a leaflet was distributed to each participant including a summarized version of the information provided in the session.

### Post-intervention Assessment

The post-intervention questionnaire was conducted immediately after the students attended the educational workshop and read the leaflet. The post-intervention assessment questionnaire included only the knowledge and attitude sections of the KAP questionnaire because salt-related behavior would require time to change. Therefore, the change in practices was assessed in the 4-week follow-up.

### Four-Week Follow-Up Assessment

After 4 weeks of the intervention session, students were invited again to complete a follow-up KAP questionnaire. The KAP questionnaire included all three components; knowledge, attitudes, and practices, and was repeated to assess the long-term impact of the educational workshop and the material provided.

### Data Analysis

Continuous data were expressed as mean ± standard deviation (SD), and categorical data were expressed as counts and percentages. Comparisons between baseline, immediate post-intervention, and 4 weeks post-intervention were conducted using the McNemar test. Scores for knowledge, attitude and practice toward salt were calculated based on the sum of the correct answers or positive attitudes/practices.

Knowledge scores ranged from 0 to 29 based on the number of correct answers, attitude scores ranged from 0 to 5 based on positive attitudes toward salt, and practice score ranged from 0 to 13 based on the number of positive practice responses (15, 16). Scores were calculated for all components in the pre-intervention and in the 4-weeks follow up. However, only knowledge and attitude scores were calculated immediately post-intervention. Comparison between knowledge scores at baseline, immediate post-intervention, and 4 weeks post-intervention was conducted using the paired sample *t*-test.

Participants were stratified based on based on their responses to seven of the practice questions to low-risk, moderate-risk and high-risk groups (22). These included “check food labels specifically for salt,” “sodium shown on the label the affects purchasing,” try to buy “low salt foods,” “try to buy ‘no added salt’ food,” “adding salt during food preparation,” “using stock cubes during cooking,” and “adding salt before tasting.” Risk scores were calculated pre-intervention and at the 4-weeks follow-up. Participants were given 1 point if they answered “always” or “often” to adding salt during food preparation, using stock cubes during cooking, and adding salt before tasting, and 1 point if they answer “never” for any of the other four practices that lead to higher salt consumption. Consequently, participants with zero points were categorized as low risk for high salt consumption, participants with 1 point as moderate risk, and with 2 or more points as high risk (22). *P* < 0.05 were considered statistically significant. Data was analyzed using SPSS software (27), version 26.0 (SPSS, Chicago, IL, USA).

## Results

### Sample Characteristics

A total of 90 students participated in the study. Key demographic variables are shown in Table 1. Participants' ages ranged from 18 to 24 years (mean = 20.7, standard deviation = 1.35), with a female to male ratio of almost 1:1 (54.4% females, 45.6% males). The majority of the participants were enrolled in applied science majors (82.2%), lived with their family (76.7%) and consumed most meals at home (76.7%). Almost half of the participants had normal BMI (48.9%) with the remaining classified as overweight and obese (21.1%, 22.2%) respectively.

**Table 1:** Characteristics of the study participants (*N* = 90).

**Characteristics**		
Age (years), mean ± SD	20.72 ± 1.35	
**Gender, n (%)**		
Male	41	(45.6)
Female	49	(54.4)
**College, n (%)**		
Applied Sciences	74	(82.2)
Humanities	16	(17.8)
**Residential type, n (%)**		
With family	69	(76.7)
Hostel	19	(21.1)
Alone	2	(2.2)
**Most meals consumed at, n (%)**		
Homemade	69	(76.7)
Restaurant	21	(23.3)
**BMI Classification (kg/m** ^2^ **), n (%)**		
Underweight (<18.5)	7	(7.8)
Normal (18.5–24.9)	44	(48.9)
Overweight (25–29.9)	19	(21.1)
Obese (≥30)	20	(22.2)

### Pre-intervention Questionnaire Responses: Knowledge, Attitude, and Practice

The number and percentage of students who answered knowledge related questions correctly is shown in Table 2. During pre-intervention, sodium percentage in salt was identified correctly by only 13.3% of the students. A high proportion answered correctly to higher salt intake and its relation to a disease risk factors; hypertension (83.3%), CVD (62.2%), water retention (67.8%) and renal diseases (65.6%). Similarly, most of the students knew that reducing the salt intake will improve the general health (83.3%) and improve blood pressure (89%). Most of the participants categorized the salt contact in the following foods correctly: instant noodle (78.9%), pickles (77.8%), cheddar cheese (73.3%), chicken cubes (73.3%), ketchup (63.3%) and tomato paste (62.2%). Whereas, less than a third answered correctly for Iranian bread (24.4%), corn flakes (21.1%) and pita bread (13.3%).

**Table 2:** Pre-intervention, post-intervention and 4-week follow-up knowledge correct responses of study participants (*n* = 90).

**Variable^a^**	**Pre-intervention n (%)**	**Post-intervention n (%)**	***P*-value^b^**	**4-weeks follow-up n (%)**	***P*-value^c^**
Percentage of sodium in salt (40%)	12 (13.3)	73 (81.1)	<0.001	77 (85.6)	<0.001
**High salt intake may increase risk factors for**					
Hypertension (Yes)	75 (83.3)	86 (95.6)	0.007	86 (95.6)	0.013
Cardiovascular diseases (Yes)	56 (62.2)	80 (88.9)	<0.001	82 (91.1)	<0.001
Diabetes (No)	48 (53.3)	62 (68.9)	0.016	57 (63.3)	0.175
Fever (No)	41 (45.6)	61 (67.8)	0.004	50 (55.6)	0.222
Water retention (Yes)	61 (67.8)	76 (84.4)	0.001	78 (86.7)	0.001
Renal diseases (Yes)	59 (65.6)	79 (87.8)	<0.001	72 (80.0)	0.029
**Reducing salt intake will improve**					
Health (Yes)	75 (83.3)	88 (97.8)	0.001	87 (96.7)	0.008
Blood Pressure (Yes)	80 (88.9)	87 (96.7)	0.065	88 (97.8)	0.039
**Sodium content in the following foods is**					
Pita bread (High)	12 (13.3)	46 (51.1)	<0.001	48 (53.3)	<0.001
Iranian bread (High)	22 (24.4)	43 (47.8)	<0.001	54 (60.0)	<0.001
Fruits (Low)	69 (76.7)	85 (94.4)	<0.001	80 (88.9)	0.019
Fresh vegetables (Low)	70 (77.8)	84 (93.3)	0.003	82 (91.1)	0.008
Canned vegetables (High)	47 (52.2)	84 (93.3)	<0.001	82 (91.1)	<0.001
Cheddar cheese (High)	66 (73.3)	88 (97.8)	<0.001	80 (88.9)	0.003
Pickles (High)	70 (77.8)	85 (94.4)	0.003	83 (92.2)	0.015
Olive oil (Low)	57 (63.3)	71 (78.9)	0.007	71 (78.9)	0.020
Basmati rice (Low)	37 (41.1)	53 (58.9)	0.009	42 (46.7)	0.522
Egyptian rice (Low)	32 (35.6)	45 (50.0)	0.053	44 (48.9)	0.065
Milk, yogurt (Low)	42 (46.7)	46 (51.1)	0.585	60 (66.7)	0.008
Salad dressing oil (High)	53 (58.9)	65 (72.2)	0.050	68 (75.6)	0.024
Ketchup (High)	57 (63.3)	74 (82.2)	0.002	76 (84.4)	0.001
Tomato paste (High)	56 (62.2)	75 (83.3)	<0.001	75 (83.3)	0.001
Red meat (Low)	27 (30.0)	47 (52.2)	0.002	36 (40.0)	0.175
Poultry (Low)	29 (32.2)	35 (38.9)	0.307	34 (37.8)	0.522
Corn flakes (High)	19 (21.1)	27 (30.0)	0.185	29 (32.2)	0.076
Chicken cubes (High)	66 (73.3)	75 (83.3)	0.064	77 (85.6)	0.035
Instant noodle (High)	71 (78.9)	86 (95.6)	0.001	80 (88.9)	0.093
Filtered water (Low)	47 (52.2)	77 (85.6)	<0.001	74 (82.2)	<0.001

a *The correct answers are provided in brackets next to each variable*;

b *Significance of post-intervention compared to pre-intervention*;

c *Significance of follow-up compared to pre-intervention. The p-values indicate the results of McNemar test*.

Regarding attitude toward salt (Table 3), 71.1% of the students assumed that they consume “just the right amount” daily and only 10% reported being concerned about amount of salt/sodium in their diet. A relatively low proportion of students agreed with the statements: “reducing added salt is important to you” (30.0%), “reducing processed foods consumption is important to you” (34.4%), and “reducing your sodium intake is important to you” (21.1%).

**Table 3:** Pre-intervention, post-intervention and 4-week follow-up for attitude related responses of study participants (*n* = 90).

**Attitude^a^**	**Pre-intervention n (%)**	**Post-intervention n (%)**	***P*-value^b^**	**4-weeks follow-up n (%)**	***P*-value^c^**
How much salt do you think you consume (Just the right amount)	64 (71.1)	36 (40.0)	<0.001	51 (56.7)	0.029
Are you concerned about the amount of salt/sodium in the diet (Yes)	9 (10.0)	24 (26.7)	0.001	11 (12.2)	0.791
Reducing added salt to foods is important to you (Agree)	27 (30.0)	48 (53.3)	0.001	33 (36.7)	0.327
Reducing consumption of processed foods is important to you (Agree)	31 (34.4)	57 (63.3)	<0.001	39 (43.3)	0.200
Reducing your sodium intake is important to you (Agree)	19 (21.1)	51 (56.7)	<0.001	29 (32.2)	0.052

a *Attitude was assessed based on a three-point Likert scale but only answers of “positive attitude” are presented*;

b *Significance of post-intervention compared to pre-intervention*;

c *Significance of follow-up compared to pre-intervention. The p-values indicate the results of McNemar test*.

Regarding practice habits as shown in Table 4, 23.3% of the students reported that they check food labels and that their purchasing decisions are affected by the information provided on the label. Moreover, <10% checked specifically for salt/sodium content and reported that their purchasing decision was affected by the salt/sodium content on the label. Only 35.6% of the students reported rarely using stock cubes during cooking. Furthermore, 67.8% reported adding salt to food at the table; moreover, 94.4% added salt during cooking. When asked about measures taken to reduce salt intake, 28.9% of the students reported trying to cut down salt before, and 34.4% of them used spices as alternatives to salt.

**Table 4:** Pre-intervention and 4-week follow-up for practice related responses of study participants (*n* = 90).

**Practice^a^**	**Pre-intervention n (%)**	**4-weeks follow-up n (%)**	***P*-value^b^**
Check food labels (Often)	21 (23.3)	27 (30.0)	0.286
Information on food labels affects purchasing decisions (Often)	21 (23.3)	19 (21.1)	0.824
Check labels specifically for salt/sodium content (Often)	8 (8.9)	13 (14.4)	0.227
Salt/sodium content on label affects purchasing decisions (Often)	7 (7.8)	11 (12.2)	0.424
Try to buy “low salt” foods (Often)	10 (11.1)	19 (21.1)	0.022
Try to buy “no added salt” foods (Often)	3 (3.3)	6 (6.7)	0.453
Add salt to food during cooking (Rarely)	5 (5.6)	16 (17.8)	0.019
Use Stock Cubes during cooking (Rarely)	32 (35.6)	36 (40.0)	0.557
Add salt to food at the table (Rarely)	29 (32.2)	42 (46.7)	0.011
Add salt before tasting the food (Rarely)	42 (46.7)	44 (48.9)	0.851
Did you try to reduce salt intake before (Yes)	26 (28.9)	41 (45.6)	0.014
Did you try to use spices to reduce salt (Yes)	31 (34.4)	45 (50.0)	0.044
Type of bottled water (low sodium)	18 (20.0)	29 (32.2)	0.052

a *Answer options for practice questions included often, sometimes, and never, only answers of positive practice are presented*;

b *Significance of follow-up compared to pre-intervention. The p-values indicate the results of McNemar test*.

### Post-intervention Questionnaire Responses: Knowledge and Attitude

Knowledge and attitude were reassessed immediately after the educational session. As shown in Table 2, there was a significant improvement in answering correctly about the percentage of sodium in salt (81.1% compared to 13.3% in the pre-intervention, *p* < 0.001). Moreover, significant increases in identifying most food items as high or low sodium food sources were evident post-intervention except for Egyptian rice, milk, salad dressing oil, poultry, corn flakes, and chicken cubes. Likewise, students were able to correctly identify the relationship of high dietary salt intake to health and disease (Table 2). As for attitude (Table 3), all five questions had significant increase in the proportion of students choosing favorable attitudes (*p* ≤ 0.001).

### Four Weeks Follow-Up Questionnaire: Knowledge, Attitudes, and Practice

As presented in Table 2, there was a significant increase in the correct answers for most knowledge related questions from baseline (pre-intervention) to the 4-weeks follow up. Percentage of sodium in salt was identified correctly by 85.6% of the students (*p* < 0.001) compared to both pre-intervention (13.3%) and immediately post-intervention (81.1%). A significantly higher proportion of students in the 4-weeks follow-up were able to relate hypertension (*P* = 0.013), CVD (*p* < 0.001), water retention (*P* = 0.001) and renal diseases (*P* = 0.029) to high salt intake when compared to the baseline. A similar significant increase was evident in correctly identifying high/low sodium food items such as pita bread, Iranian bread, canned vegetables, ketchup, tomato paste, chicken cubes and filtered water (*p* < 0.001) (Table 2).

The proportion of students reporting positive attitudes toward salt dropped in the 4-weeks follow-up compared to the immediate post-intervention but remained above baseline in four of the attitude questions (Table 3); “concerned about the amount of salt/sodium in the diet,” “it is important to reduce added salt to foods,” “it is important to reduce consumption of processed foods,” and “reducing your sodium intake is important to you.” A significantly less proportion of students reported that they think they consume “just the right amount of salt” in the 4-weeks follow-up compared to baseline (*P* = 0.029).

Regarding practice (Table 4), there was a significant improvement in positive practices toward salt in the 4-weeks follow-up compared to the baseline for; trying to buy low-salt food (*P* = 0.022), rarely adding salt during cooking (*P* = 0.019), rarely adding salt to food at the table (*P* = 0.011), tried to reduce salt intake before (*P* = 0.014), and trying to use spices to reduce salt (*P* = 0.044). The remaining practices showed an increase in positive responses; however, no significant improvement was found between the pre-investigation and the 4-weeks follow-up.

### Knowledge, Attitude, and Practice Scoring and Risk Stratification

**Figure 2:**
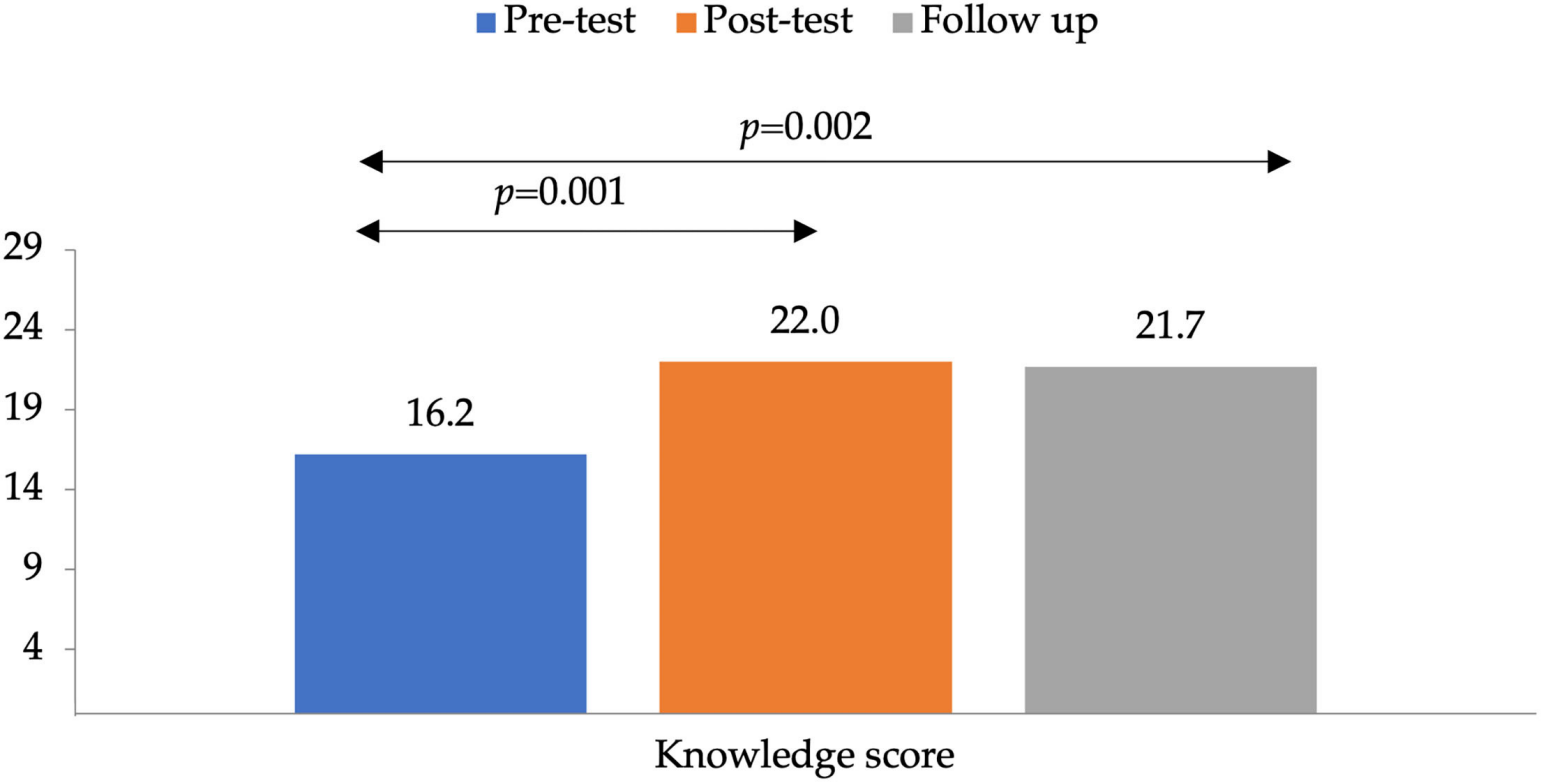
Mean knowledge scores of pre-test, post-test, and follow up. The *p*-value indicates the statistical significance of the paired sample *t*-test.

The average knowledge score at baseline was 16.2 ± 6.1 (possible scores: 0 to 29). It increased significantly to 22.0 ± 3.7 at the immediate post-intervention assessment (*P* = 0.001) and then declined to 21.7 ± 4.0 at the 4-weeks follow-up assessment, however remained significantly higher than baseline (*P* = 0.002) as shown in Figure 2. A similar trend was observed for attitude scores, where the average attitude score pre-intervention was 1.7 ± 1.2 (possible scores: 0 to 5), increased to 2.4 ± 1.5 immediately post-intervention and dropped to 1.6 ± 1.4 after 4-weeks. Moreover, the practice score increased from 2.8 ± 2.5 (possible scores: 0 to 13) pre-intervention to 3.9± 2.8 after 4-weeks (*P* = 0.001).

**Figure 3:**
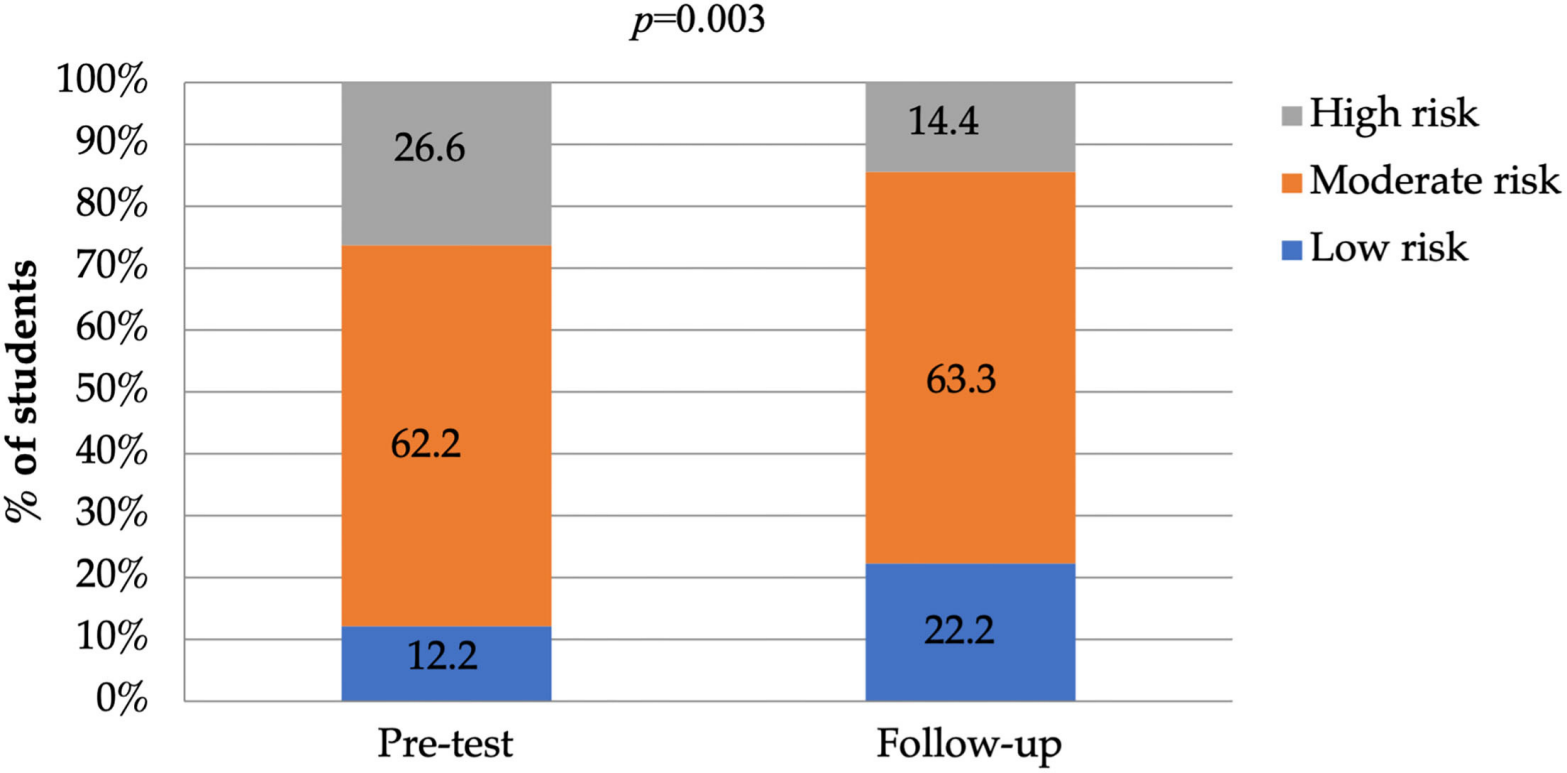
Risk of high-salt consumption categories for student's pre-test and at 4-weeks follow up (low risk, moderate risk, and high risk). The *p*-value indicates the statistical significance of the McNemar test.

At baseline, 26.6% of the students were categorized as having high risk for salt consumption practices, however, this dropped to 14.4% at the 4-weeks follow-up assessment (Figure 3). Moreover, at baseline, 12.2% of the students were categorized as having low risk behavior, and this increased to 22.2% at the 4-weeks follow-up. The McNemar test showed a trend toward practice category improving from baseline to the 4-weeks follow-up assessment (*P* = 0.003).

## Discussion

The aim of this study was to determine the efficiency of applying evidence-based nutrition intervention on salt related knowledge, attitudes, and practices among non-medical students at the University of Sharjah using a validated multi-component questionnaire and an educational interactive session. Parameters were measured before and after the intervention to allow comparison. The results revealed that salt-related knowledge improved significantly immediately post-intervention, but it was the knowledge was not fully retained until the 4-weeks follow-up, however it remained above the baseline. Moreover, improvement in the high-risk for salt consumption group was recorded, as a shift was observed toward the moderate-risk group and a trend toward practice category improving from baseline to the follow-up was shown.

Findings at baseline showed a relatively low salt/sodium related knowledge with low tendency toward implementing salt/sodium reduction behaviors. These findings were consistent with the previous study among the UOS students (16). For example, the mean knowledge score in this study was 16.2 ± 6.1 compared to 17.2 ± 6.1 in the previous study (16). Also consistent with the results of health science students in Bangladesh on salt related knowledge (28). Moreover, more than half of the students in the current study estimated that they consume just the right amount of salt. Similarly, 62% of adults in the UAE reported consuming the right amount of sodium (15). However, the same study estimated that more than two-thirds of the participants exceeded the WHO recommendations (15). These findings suggest that perceived intake does not necessarily reflect actual intake. Most of the students were aware of the obvious adverse health effects associated with high salt intake. Similar findings were reported in several studies in the UAE, European countries and the Lebanon investigating salt related knowledge, attitude, and behavior (15, 16, 28, 29). Also, more than three in four students indicated that salt reduction would actively improve their health and blood pressure which was in contrast to a study reported among adults in Montenegro where less than half of the participants had the same indication (29).

Furthermore, findings showed that less than a quarter of the participants check food labels and use the information on food labels to guide their purchasing decisions. More worryingly, a very small proportion of students check for salt content on the food label and use it to guide their food choices. In the previous study among UOS students only 17% reported checking food labels and 20% of students used the labels to guide their purchasing decisions (16). Findings in a study among adult consumers in Lebanon revealed much lower proportion for checking food labels than those reported in the UAE; stating that food labels are not adequately utilized by participants (30). Therefore, an awareness of the health risks associated with high salt consumption is recommended to increase salt label usage and purchases of low salt foods.

With respect to knowledge, in all questions, the percentage of correct answers of the immediate post-intervention was significantly better compared to the pre-intervention (baseline). After 4 weeks follow-up, the percentage of correct answers showed that retention of knowledge of students slightly decreased in comparison to the immediate post-intervention, however remained above baseline. A similar pattern was seen in a previous study conducted in Lebanon (22). Similarly, a cohort study conducted at Lurio University, Mozambique, evaluating knowledge retention showed a decrease in knowledge after 6 months compared to post-test (31). Despite the difference in the follow up time intervals, the general trend of decline in knowledge is consistent. Moreover, historically a similar decay in knowledge-retention over time following an educational program is shown in other studies (32, 33).

Due to the fact that knowledge is an important driver of attitudes and practices, it is expected that a decline in knowledge will be accompanied by similar drop in favorable attitude responses (34). As it is evident in our results, after 4 weeks there was a decrease in the positive attitude response compared to baseline simultaneously with the decline in knowledge, however knowledge remained above baseline. Therefore, conducting frequent periodical educational sessions along with testing knowledge has been used effectively to ensure long-term knowledge retention (35, 36). Aligning with our study, such strategy where students are educated on aspects of knowledge and followed-up must be implemented.

Several salt related practices showed significant improvements after the 4 weeks follow up, such as trying to purchase low-salt food and rarely adding salt to food at the table. However, no significant improvement was found in other salt-related practices such as using stock cubes during cooking and adding salt before tasting the food. The results are consistent to what was found in the intervention study performed in Iran, in which it was shown a significant increase in the mean and standard deviation of KAP among the intervention group, and a significant decrease in the mean salt intake (37). Nonetheless, in a study investigating the impact of community-based salt reduction program in Australia, the proportion of participants reporting salt reducing practices such as avoiding processed foods and checking food labels decreased significantly (38).

Our results indicate a trend toward an improve in the salt-related practices from baseline to the 4-weeks follow-up such as trying to buy low-salt food, rarely adding salt during cooking or at the table and trying to reduce salt intake by using spices. Evidence suggests that intervention strategies including peer group education has shown similar positive impact on salt-related practices and salt consumption. A study among adults having at least one risk factor of CVD indicated beneficial effects of peer group intervention on cardiovascular risk factors, with significant improvements in total score and more specifically on tobacco cessation (34). Moreover, a national consumer awareness campaign about the negative effect of salt on health in the United Kingdom has shown promising results as it indicated a significant decline in using salt at the table (39). Another randomized clinical trial depicted a lower dietary sodium intake among the intervention group (40).

There are several limitations to this study. The findings may not be generalized to all young adults in the UAE as our study was restricted to the non-medical major university students. Including participants who showed an interest in the study (self-selection) is likely to create a group of more motivated persons which could affect sample representativeness. In addition, the questionnaire included the use of self-reported attitudes and practices, that possibly might cause some respondent bias or misreporting of data that may not accurately reflect actual attitudes and practices. Another limitation to this study was the use of a single contact intervention due to time constraint. Hence, future studies with multiple interactions are encouraged to induce long-term benefits.

Our study showed a significant increase in knowledge and positive salt related attitude immediately post-intervention. This increase remained significant after the 4-weeks follow-up. However, there was a tendency to have a slight decline in knowledge after 4-weeks as it was only a single contact. A complementary educational method is advised to enhance retention of knowledge and probably have an impact on salt-related attitudes and practices. On the other hand, it was evident that changes in practice were not as prominent as changes in knowledge and attitude. This is possibly related to poor dietary habits and high consumption of fatty food, snacks, sugar, and fast food that is evident in this age group.

In conclusion, it is crucial to conduct several specifically designed awareness sessions and space them over time in conjunction with creative intervention programs focusing on the proper way of reading and using food and salt labels. Moreover, it is suggested to deliver nutrition related message using social media, given that this generation is most influenced by these platforms. It is also suggested to mandate at least one nutrition education course for all university students as well as ensuring the provision of a wide variety of healthy food options with low salt content at the university campus cafeteria and healthy vending machine snacks. Future research should also focus more on a long-term knowledge retention to investigate the impact of salt-reduction interventions on related attitude, practice, and overall health. Conducting similar studies on a large scale is recommended to be able to generalize results to the young adult population.

## Data Availability Statement

The datasets presented in this study can be found in online repositories. The names of the repository/repositories and accession number(s) can be found at: FigShare: 10.6084/m9.figshare.15062367.

## Ethics Statement

The studies involving human participants were reviewed and approved by the University of Sharjah Research Ethics Committee (UOS-REC) reference number: REC-19-04-15-01-S. The patients/participants provided their written informed consent to participate in this study.

## Author Contributions

LC, MH, AJ, MM, and AA: conceptualization and methodology. LC, MM, and SS: formal analysis. LC, MH, RAD, RAR, SA, AA, SQ, and FM: investigation. LC, SS, MM, and AA: writing—original draft preparation. LC, MH, AJ, MM, RAD, RAR, SA, AA, SQ, FM, SS, LS, and AA: writing—review and editing. MM: visualization. LC: supervision. All authors have read and agreed to the published version of the manuscript.

## Conflict of Interest

The authors declare that the research was conducted in the absence of any commercial or financial relationships that could be construed as a potential conflict of interest.

## Publisher's Note

All claims expressed in this article are solely those of the authors and do not necessarily represent those of their affiliated organizations, or those of the publisher, the editors and the reviewers. Any product that may be evaluated in this article, or claim that may be made by its manufacturer, is not guaranteed or endorsed by the publisher.
