# Supplementary material for: Impact of a Nutrition Education Intervention on Salt/Sodium Related Knowledge, Attitude, and Practice of University Students
Source: Front Nutr. 2022 Feb 25;9:830262. doi: 10.3389/fnut.2022.830262 (PMC8914224; doi:10.3389/fnut.2022.830262)
Supplement: Supplementary file 1 [file Data_Sheet_1.docx]

Supplementary Material - Study Questionnaire

**Impact of Nutrition Education Intervention Regarding Salt/Sodium Related Knowledge, Attitude & Practice on University Students**

| **Participant Code:** |  | | | | |
| --- | --- | --- | --- | --- | --- |
| **College:** |  | | | | |
| **Age:** |  | | | | |
| **Weight (kg):** | | | **Height (cm):** | | **BMI (kg/m²):** |
| **Blood Pressure** | | **Systolic (mmHg):** | | **Diastolic(mmHg):** | |

1. **Gender?**

□ Male

□ Female

1. **College?**

□ Medical & Health Sciences (College of Medicine, Dentistry, Pharmacy, Health Sciences)

□ Applied Sciences (College of Engineering, Sciences, Business)

□ Humanities (College of Law, Sharia, Social Sciences, Communication, Fine Arts)

1. **Residence?**

□With family/relatives

□ University hostel

□ Alone

1. **Most of your meals are?**

□ Homemade

□ Restaurants

1. **Knowledge scale of participants**

**1. How much the percentage of sodium in salt?**

□ 20%

□ 40%

□ 60%

□ I don’t know

|  | **Sodium content in the following foods considered** | **Low** | **High** | **I don’t know** |
| --- | --- | --- | --- | --- |
| 2 | Pita bread |  |  |  |
| 3 | Iranian bread |  |  |  |
| 4 | fruits, considered |  |  |  |
| 5 | Fresh vegetables |  |  |  |
| 6 | Canned vegetables |  |  |  |
| 7 | Cheddar cheese |  |  |  |
| 8 | Pickles |  |  |  |
| 9 | Olive oil |  |  |  |
| 10 | Basmati rice |  |  |  |
| 11 | Egyptian rice |  |  |  |
| 12 | Milk, yoghurt |  |  |  |
| 13 | Salad dressing oil |  |  |  |
| 14 | Ketchup |  |  |  |
| 15 | Tomato paste |  |  |  |
| 16 | Red meat |  |  |  |
| 17 | Poultry |  |  |  |
| 18 | Corn flacks |  |  |  |
| 19 | Chicken cubes |  |  |  |
| 20 | Indomi noodle’s |  |  |  |
| 21 | Water, filtered |  |  |  |
|  | **High salt intake may increase risk factors for….** | Yes | No | I don’t know |
| 22 | Hypertension |  |  |  |
| 23 | Cardiovascular diseases |  |  |  |
| 24 | Diabetes |  |  |  |
| 25 | Fever |  |  |  |
| 26 | Water retention |  |  |  |
| 27 | Renal diseases |  |  |  |
|  | **If I reduce salt intake this will……** | Yes | No | I don’t know |
| 28 | Improve my health |  |  |  |
| 29 | Improve my blood pressure |  |  |  |

1. **Attitude toward salt in the last 3 months**

**1. How much salt do you think you consume?**

□ Too much □ Just the right amount □ Far too little

**2. Are you concerned about the amount of salt/sodium in your diet?**

□ Yes □ No □ I don’t know

**3. Reducing the amount of salt you add to foods is definitely important to you?**

□ Disagree □ Neutral □ Agree

**4. Reducing the amount of processed foods you eat is definitely important to you?**

□ Disagree □ Neutral □ Agree

**5. Reducing your sodium intake is definitely important to you?**

□ Disagree □ Neutral □ Agree

1. **Practice toward salt in the last 3 months**

**1. Do you Check food labels?**

□ Rarely □ Sometimes □ Often

**2. The information on food labels affects purchasing decisions?**

□ Rarely □ Sometimes □ Often

**3. Do you check labels specifically for salt/sodium content?**

□ Rarely □ Sometimes □ Often

**4. Salt/sodium content indicated on label affects purchasing decisions?**

□ Rarely □ Sometimes □ Often

**5. Do you try to buy “low salt” foods?**

□ Rarely □ Sometimes □ Often

**6. Do you try to buy “no added salt” foods?**

□ Rarely □ Sometimes □ Often

**7. Do you add salt to food during preparation or cooking?**

□ Rarely □ Sometimes □ Often

**8. Do you use Stock Cubes during cooking?**

□ Rarely □ Sometimes □ Often

**9. Do you add salt to food at the table?**

□ Rarely □ Sometimes □ Often

**10. Do you add salt before tasting your food?**

□ Rarely □ Sometimes □ Often

**11. Did you try to reduce salt intake before?**

□ Yes □ No □ I don’t know

**12. Did you try to use spices to reduce salt intake?**

□ Yes □ No □ I don’t know

**13. Which bottled water do you drink most of the time?**

□ Regular filtered water (masafi, alain, arwa, etc...)

□ Low sodium filtered water

□ I don’t know
